# Role of the major histocompatibility complex class II protein presentation pathway in bone immunity imbalance in postmenopausal osteoporosis

**DOI:** 10.3389/fendo.2022.876067

**Published:** 2022-08-11

**Authors:** Xiaoning Wang, Xin Zhang, Yidan Han, Xinwei Duan, Jianchang Wang, Hui Yan, Shanshan Wang, Yunteng Xu, Zaishi Zhu, Lili Wang, Yanfeng Huang, Qing Lin, Xue Tan, Junkuan Zhuo, Haifeng Zhang, Min Mao, Weiying Gou, Zhouping Yi, Xihai Li

**Affiliations:** ^1^ College of Integrative Medicine, Fujian University of Traditional Chinese Medicine, Fuzhou, China; ^2^ Academy of Integrative Medicine, Fujian University of Traditional Chinese Medicine, Fuzhou, China; ^3^ Fujian Key Laboratory of Integrative Medicine on Geriatrics, Fujian University of Traditional Chinese Medicine, Fuzhou, China

**Keywords:** postmenopausal osteoporosis, MHC-II molecule, protein presentation pathway, effector T cells, bone immunity

## Abstract

Bone immunity regulates osteoclast differentiation and bone resorption and is a potential target for the treatment of postmenopausal osteoporosis (PMOP). The molecular network between bone metabolism and the immune system is complex. However, the molecular mechanism underlying the involvement of the major histocompatibility complex class II (MHC-II) molecule protein presentation pathway in PMOP remains to be elucidated. The MHC-II molecule is a core molecule of the protein presentation pathway. It is combined with the processed short peptide and presented to T lymphocytes, thereby activating them to become effector T cells. T-cell-derived inflammatory factors promote bone remodeling in PMOP. Moreover, the MHC-II molecule is highly expressed in osteoclast precursors. MHC-II transactivator (CIITA) is the main regulator of MHC-II gene expression and the switch for protein presentation. CIITA is also a major regulator of osteoclast differentiation and bone homeostasis. Therefore, we hypothesized that the MHC-II promotes osteoclast differentiation, providing a novel pathogenic mechanism and a potential target for the treatment of PMOP.

## Introduction

Postmenopausal osteoporosis (PMOP), a major public health concern, is attributed to an imbalance of bone metabolism in postmenopausal women. The condition is characterized by decreasing bone strength and bone mineral density (1–3). There are several pathological mechanisms involved in the development of PMOP, such as imbalances in osteogenic/adipogenic differentiation of bone marrow mesenchymal stem cells (BMSCs), osteogenic and/or osteoclast (OC) coupling, and bone immunity. Among them, bone immunity imbalance has recently attracted more attention.

The relationship between bone metabolism disorder and the immune system is complex (4). Major histocompatibility complex class II molecules (MHC-II) play essential roles in the adaptive immune response and participate in the immune regulation of bone health (5–7). However, the molecular mechanism underlying the regulatory effects of MHC-II on bone metabolism imbalance remains unclear. Therefore, this review summarizes the currently available knowledge regarding this molecular mechanism. The following aspects are discussed. Activation of T and B lymphocytes requires MHC-II molecules, and loss of estrogen (E2) leads to the conversion of T cells to effector T cells (T_E_) and the chronic production of related inflammatory cytokines. In turn, T_E_ promote bone remodeling by releasing inflammatory factors. The main cytokines produced by T_E_ affect bone resorption and bone formation. MHC-II gene expression is inseparable from the regulation of MHC-II transactivator (CIITA) and phagolysosomal membrane integrity. Moreover, bioinformatics revealed the potential pathologic association between PMOP and T activation, as well as the protein presentation pathway. Understanding the mechanisms through which MHC-II molecules regulate bone homeostasis may facilitate the development of targeted drugs for the treatment of PMOP.

There are two types of MHC molecules involved in adaptive immune response in mammals, namely, MHC-I and MHC-II. The former is distributed in almost all nucleated cells in the body; the latter is distributed in professional antigen-presenting cells (APCs), such as monocytes/macrophages, dendritic cells (DCs), and B cells. Both types are tightly regulated and involved in the activation process of effector T lymphocytes (T_E_) (8–10).

## MHC-II molecules as key immune molecules participating in bone remodeling

### OC precursors highly express MHC-II molecules

As OC precursors, monocytes/macrophages are a type of professional APCs. Hence, it is more meaningful to investigate the effect of the MHC-II molecule protein presentation pathway on bone metabolism in PMOP.

### Activation of T and B lymphocytes requires MHC-II molecules

How can the initial T cells and memory T cells (T_M_) be activated and induce inflammation? A series of processes are required for T cells to induce adaptive immune responses and inflammation. The initial activation of T cells and T_M_ is followed by further proliferation and differentiation into T_E_. The activation of the initial T cells and T_M_ requires signals from the interaction between the T-cell receptor and the MHC-antigenic peptide complex. MHC molecules monitor different proteolytic machineries in APCs and transport the hydrolyzed peptide to the surface of APCs for the identification and combination of initial T cells and T_M_ (11–13). DCs are the most powerful APCs in the body, particularly mature DCs whose main feature is the high expression of MHC-II molecules. Therefore, the MHC-II molecular presentation pathway plays an important role in activating the initial T cells and T_M_ to become T_E_ and may be closely related to the pathogenesis of PMOP.

It is well established that extracellular proteins enter the APCs and participate in the formation of phagosomes. Phagosomes combine with intracellular lysosomes to form phagolysosomes, where proteins are hydrolyzed into short peptides. MHC-II molecule–peptide complexes migrate to the surface of APCs for identification by CD4^+^ T cells, thereby activating them to become T helper 1 (Th1), Th2, Th17, etc. It is established that Th2 provides CD40L for the activation of B lymphocytes. Activated T and B lymphocytes can produce interferon-gamma (IFN-γ), tumor necrosis factor-alpha (TNF-α), interleukin-17A (IL-17A), and receptor activator of nuclear factor-κB (NF-κB) ligand (RANKL) (14), which are important immune molecules involved in bone remodeling in PMOP. This evidence further shows that MHC-II molecules are closely related to the occurrence and development of PMOP.

## E2 deficiency leads to conversion of T cells into T_E_ and chronic production of related inflammatory cytokines

Recently, a new pathway has been described, indicating that E2 loss results in chronic production of TNF-α and IL-17 by converting T_M_ into T_E_. IL-7 and IL-15 are involved in the process; both are mainly secreted by bone marrow dendritic cells (BMDCs), which are important APCs in the bone marrow (BM) (15). Animal experiments have shown the absence of bone loss in specially treated mice, in which T_M_ cannot convert into T_E_ (16).

Physiologically, E2 can induce apoptosis of BMDCs and T_M_ through the Fas ligand pathway (15). In the absence of E2, BMDCs exist for a prolonged period of time. This effect leads to antigen-independent activation of T_M_ to produce TNF-α and IL-17A (16). In general, the classical activation and conversion of T_M_ into T_E_ require antigen stimulation (17). However, the activation induced by loss of E2 differs from that model (15), and this difference may be associated with chronic inflammation in PMOP. We hypothesize that, in postmenopausal women, the lifespan of DCs and T_M_ is extended with the decline in E2. This further contributes to the conversion of initial T cells and T_M_ activation into T_E_, resulting in the release of various inflammatory factors and causing PMOP.

## T_E_ promotes bone remodeling by releasing inflammatory factors

The adaptive immune system is fundamental to the progression of PMOP (15). Adaptive immune responses are composed of cellular immunity and humoral immunity. The former is mainly mediated by T lymphocytes, while the latter is mainly mediated by B lymphocytes.

The deficiency of E2 promotes persistent inflammation, which is conducive to the development of PMOP. Mechanistic studies on the relationship between menopausal E2 loss and activation of T cells have primarily been performed in rodents with ovariectomy (OVX); the key results obtained from these investigations have been verified in human studies (15). Bone loss was decreased in T-cell-deficient mature mice with OVX, demonstrating that T cells are required to promote bone resorption in PMOP (18–22). T_E_ can secrete numerous cytokines, such as IFN-γ, TNF-α, and IL-17A.

### Main cytokines produced by T_E_ affect bone resorption

OCs are multinucleated giant cells formed by the fusion of multiple mononuclear macrophages differentiated from myeloid progenitor cells in the BM. OCs appear to be sensitive to cytokines produced by T_E_, such as IFN-γ, TNF-α, and IL-17A.

IFN-γ derived from T_E_ can regulate the RANKL signal pathway during OC differentiation (20). Th1 cells are one of the main T_E_ in T-cell immunity and the major producers of IFN-γ. Initially, bone loss due to inflammation was attributed to a Th1-mediated pathological process. However, it was later demonstrated that Th17 cells are the main drivers of bone loss (23).

It has been reported that mature monocytes/macrophages differentiate into OCs in an IFN-γ-rich microenvironment and promote cell fusion (24). Moreover, IFN-γ could readily induce monocyte aggregation, leading to the formation of multinuclear giant cells (25). In addition, it has been shown that TNF-α can directly act on OCs and their precursors, and it cooperates with the RANKL signal pathway in osteoclastogenesis (26–29). TNF-α can recruit TNF receptor-associated factors to sequentially activate NF-κB p50, and, c-Fos, and nuclear factor of activated T cells 1 (NFATC1) for the promotion of OC differentiation. This process is similar to and independent of the RANKL pathway (30). Blockade of the pathways by which lymphocytes migrate to the BM reduced the levels of TNF-α and Th17 cells in the BM after OVX in mice. These effects were accompanied by trabecular bone loss in this model (31).

E2 deficiency can increase the number of Th17 cells and TNF-α-producing T cells in the BM, and this process is dependent on the gut microbiome. Subsequently, BM IL-17A and TNF-α stimulate RANKL expression and activity, causing bone loss. Demonstrating the functional relevance of T-cell trafficking, blockade of Th17 cells and TNFα-producing T cells from the gut or their influx into the BM prevented OVX-induced bone loss. Therefore, it can be concluded that T cells in the gut are proximal targets of E2 deficiency-induced bone loss in PMOP (32).

Additionally, as an immune cytokine, IL-17A participates in the regulation of bone remodeling (15). IL-17A is mainly secreted by a special subtype of T_E_, namely, Th17 cells (33), and promotes bone destruction (34–36). Of note, there is a one-quarter amino acid sequence homology of IL-17A between humans and mice (37). It is thought that IL-17A participates in inflammation and may be mainly derived from the activated memory CD4^+^ T cells (T_M_), which subsequently differentiate into Th17 cells (37, 38). The local cytokine environment can promote or protect against bone loss. In addition, the mechanisms through which TNF-α and IL-17A affect bone metabolism *via* OCs have been studied extensively (39, 40).

IL-17A signaling plays a role through the IL-17A receptor (IL-17AR); however, the role of IL-17A signaling in OCs remains elusive (41). Different concentrations of IL-17A exert varied effects on OCs. A low concentration of IL-17A (0.5 ng/ml) can promote OC differentiation *via* RANKL-JUN N-terminal kinase (RANKL-JNK) signaling and reduce the apoptosis of OCs through the RANKL–beclin 1 (BECN1)–autophagy–TRAF3 pathway. IL-17A increases the number of OC precursors to influence subsequent RANKL-dependent OC differentiation. However, a high concentration of IL-17A (5–50 ng/ml) could inhibit OC differentiation and stimulate the apoptosis of OCs *via* the two aforementioned pathways (42, 43). Interestingly, a higher concentration of IL-17A (100 ng/ml) increases the number of OC precursors and induces OC formation (34). IL-17A also indirectly targets the OC-supporting cells, such as BMSCs, osteoblasts (OBs), and osteocytes, to produce various cytokines and molecules for the regulation of OC differentiation (4). Binding of IL-17A to its receptor IL-17AR on pre-OC triggers Act1 adaptor protein and may activate the downstream Janus kinase 2-signal transducer and activator of transcription 3 (JAK2-STAT3) signal, which can promote RANKL expression (44–47). The upregulation of RANKL and the increase in the RANKL/osteoprotegerin ratio could promote OC differentiation.

Theories on the activity of IL-17A in OC differentiation remain controversial; thus, it is imperative to explore the specific underlying mechanisms (48). Specific subtypes of T_E_ express TNF-α, which increases OB apoptosis and indirectly stimulates osteoclastogenesis *via* B-cell-produced RANKL, thereby triggering bone loss during PMOP (49).

### The main cytokines derived from T_E_ show different effects on bone formation

As discussed above, inflammation affects bone resorption. However, studies on the role of inflammatory factors in restraining bone formation are currently limited. Physiologically, under coupled bone remodeling conditions, there is a dynamic balance between bone resorption and formation. For example, increasing resorption is accompanied by the recruitment of BMSCs and their conversion into OBs (15). However, this process appears to be impaired in the presence of inflammatory cytokines (i.e., TNF-α and IL-17). Therefore, bone formation is reduced versus bone resorption, which is consistent with the pathological mechanism of PMOP. OBs and BMSCs are sensitive to TNF-α and IL-17A.

TNF-α can inhibit bone formation by suppressing OB differentiation. It can inhibit the expression of osterix (OSX) and runt-related transcription factor 2 (RUNX2), which are vital to OB differentiation (50, 51). RUNX2 is a specific transcription factor that could commit BMSCs to the OB pathway. It has been demonstrated that TNF-α could restrict the differentiation of BMSCs into OBs by regulating RUNX2 expression (15). OSX is another key transcription factor for OB maturation, and TNF-α also can target OSX expression (52–54). Furthermore, OB differentiation can be regulated *via* the mechanistic target of rapamycin (mTOR) pathway (55–58). Studies have shown that TNF-α can preferentially regulate cellular metabolism in adipocytes and muscle cells (59–61). OBs, adipocytes, and muscle cells originate from BMSCs through different directions of differentiation. The mechanism by which TNF-α regulates OB cellular metabolism is currently unknown. *In-vitro* studies have shown that TNF-α can regulate autophagy and apoptosis *via* the NF-κB signal pathway in OBs (62–64), both of which are controlled by the mTOR.

In ankylosing spondylitis, a study on human pre-OB has indicated that IL-17A could promote bone-derived cells to differentiate into OBs through the JAK2/STAT3 signal pathway (65). IL-17A can promote the differentiation of BMSCs into OBs and the mineralization of OBs by upregulating the expression of bone formation-related gene alkaline phosphatase and RUNX2 (66). IL-17A and bone morphogenetic protein 2 (BMP2) could promote the osteogenic differentiation of BMSCs (67). OBs and adipocytes are both differentiated from a common pluripotent precursor, namely, BMSCs. The decision for the differentiation of BMSCs into OBs or adipocytes is delicately balanced and there is competition. IL-17A may steer BMSCs into OBs. Moreover, it can activate cyclooxygenase 2 (COX2)-induced prostaglandin E2 (PGE2) to inhibit lipid-related proteins, such as peroxisome proliferator-activated receptor gamma (PPARγ) and adiponectin. This process leads to a reduction in the differentiation of BMSCs into adipocytes (68). Therefore, IL-17A may exert different effects on OCs and OBs and can induce extensive bone turnover in PMOP.

It has been demonstrated that IL-17A could affect the differentiation of BMSCs into OBs and the functions of mature OBs (40). Th17 cells release IL-17A, which directs mesenchymal stem cell differentiation toward the osteogenic lineage but also indirectly increases OC differentiation (49).

In summary, bone metabolism is sensitive to chronic inflammation induced by the activation of T cells in PMOP. Specifically, deficiency of E2 promotes the conversion of T_M_ into T_E_ in the BM.

## Key aspects of MHC-II molecule protein complexes presented to T cells

Proteins enter APCs and participate in the formation of phagosomes. Phagosomes combine with intracellular lysosomes to form phagolysosomes, where proteins are hydrolyzed into short peptides. Meanwhile, MHC-II molecules synthesized in the endoplasmic reticulum are transported to MHC class II-containing compartments, where peptides are loaded in the peptide-binding groove of MHC-II molecules (69). Phagolysosomes associate with MHC class II-containing compartments to form terminal lysosomes in APCs. MHC-II molecule–peptide complexes migrate to the cell surface for identification by CD4^+^ T cells and activation of adaptive immune responses. Therefore, the expression of MHC-II molecules and the integrity of the phagolysosomal membrane are critical for the presentation of the MHC-II molecule protein complexes to T cells.

### Expression of MHC-II genes is dependent on the regulation of CIITA

It has been shown that CIITA is a master regulator of MHC-II genes in APCs, which are critical for the activation of T cells and the induction of adaptive immune response (8, 9).

Under physiological conditions, CIITA is the master regulator of MHC-II genes (8, 9). It is a non-DNA-binding co-activator, which can specifically regulate the expression of MHC-II molecules. CIITA deficiency could result in rare human immunodeficiency disease (70). Overexpression of CIITA induces severe spontaneous osteoporosis by an increase in the number of OCs and bone resorption (7).

The classical MHC-II molecules in humans (HLA-DR, HLA-DP, and HLA-DQ) are the major target genes of CIITA. The factors which regulate MHC-II expression play roles *via* the promoters that drive transcription of the MHC2TA gene encoding CIITA. As OC precursors, macrophages constitutively and highly express MHC-II molecules (8, 9). In the presence of low levels of CIITA, the synthesis of MHC-II molecules can be limited, and the presentation of MHC-II molecule proteins in DCs (a type of APC) is impaired (18). The expression of genes which encode accessory proteins required for MHC-II molecule protein presentation can also be regulated by CIITA. Thus, CIITA is a central regulator controlling the response to proteins that will be processed and the maintenance of tolerance in the immune system (8, 9). This coordinated regulation of MHC-II and other genes necessary for its function is unique; hence, CIITA has been termed the “master regulator” of MHC-II molecules and the protein presentation pathway (8, 9).

Therefore, it is hypothesized that overexpression of CIITA will lead to overexpression of MHC-II molecules, which in turn can cause overactivation of the protein presentation pathway. Subsequently, more initial T cells and T_M_ are activated to become T_E_, resulting in excessive immune response and inflammation in PMOP. As monocytes/macrophages are a type of professional APCs, we hypothesized that the relationship between CIITA and MHC-II may be in the OC precursors.

### Phagolysosomal membrane integrity determines whether MHC-II molecule–peptide complexes can be presented

Phagolysosome is a critical endocytic organelle in the MHC-II molecular protein presentation pathway. Its membrane integrity determines whether MHC-II molecule–peptide complexes can be presented. The rupture of the membrane before the presentation of the MHC-II molecule–peptide complexes will lead to APC death and lack of T-cell activation.

Following the completion of the MHC-II molecule–peptide complexes, the membrane of the phagolysosome fuses with the cell membrane. At the same time, the peptide-binding region of the MHC-II molecule can bind to processed peptides, and its immunoglobulin-like region can be specifically recognized by CD4 molecules expressed on T cells.

The effect of phagolysosomal membrane integrity on the MHC class II molecular protein presentation pathway is critical. However, the mechanism regulating the integrity of the phagolysosomal membrane is unknown. The maintenance of phagolysosomal membrane integrity is regulated by numerous factors, such as the osmotic control of membrane tension, lipid bilayer modifications and renitence vacuoles, and membrane-stabilizing proteins (71).

The membrane-stabilizing proteins are of particular interest. It has been reported that the Bin–amphiphysin–Rvs (BAR) domain-containing protein family plays important roles in scaffolding and stabilizing the curved membranes (72). These proteins will increase the surface of the endocytic organelle, thereby facilitating the rapid export of osmolytes that can diffuse into the tubules and access the membrane solute carriers (73, 74).

Mature endocytic organelles, including phagolysosomes, require transmembrane proteins to protect the membrane from the harsh luminal environment (75, 76). The lysosome-associated membrane proteins (LAMP1 and LAMP2) and the lysosome integral membrane protein 2 (LIMP2) have been well investigated (76, 77). Loss of LAMP1 and LAMP2 does not affect the lysosomal membrane integrity (78, 79). In contrast, loss of LIMP2 results in severe damage to the lysosomes (76). The homolog of the human LIMP2, SCAV-3, has been identified as an important regulator of lysosome integrity (76). Loss of SCAV-3 can lead to the rupture of lysosome membranes. Therefore, SCAV-3 is vital for preserving lysosomal membrane stability. Notably, modulation of lysosome integrity by the insulin/insulin-like growth factor 1 (insulin/IGF1) signaling pathway affects longevity (76).

In summary, we conclude that phagolysosomal membrane integrity determines whether MHC-II molecule–peptide complexes can be presented to T cells. This affects their activation and conversion into T_E_, thereby influencing the release of numerous cytokines and the development of PMOP.

## MHC-II may promote OC differentiation

It has been demonstrated that CIITA is a key regulator of OC differentiation and bone remodeling (6). Previous studies have shown that CIITA exerts an indirect effect on bone homeostasis during E2 deficiency-induced bone loss, which may be associated with its effects on protein presentation. In OVX mice, an increase in the expression of CIITA increased the expression of MHC-II molecules and enhanced activation-induced T-cell proliferation (75). Hence, it is vital to activate T cells for bone loss caused by OVX, and CIITA could be regulated by the presence of E2. The regulation of CIITA is dependent on IFN-γ, because OVX leads to increased levels of IFN-γ derived from T_E_. Of note, it has been shown that CIITA was not upregulated in IFN-γR-/p-mice (80). Therefore, it would be meaningful to examine the expression of CIITA in OC precursors during PMOP.

CIITA is a key regulator of the activation of T cells and should be considered an important factor in the relationship between the immune response and bone health (7). However, the effect of the MHC-II molecule on OC differentiation is currently unknown. We hypothesize that MHC-II molecules could also promote OC differentiation.

## Bioinformatics revealed a potential pathologic association between PMOP and T activation, as well as the protein presentation pathway

**Figure 1:**
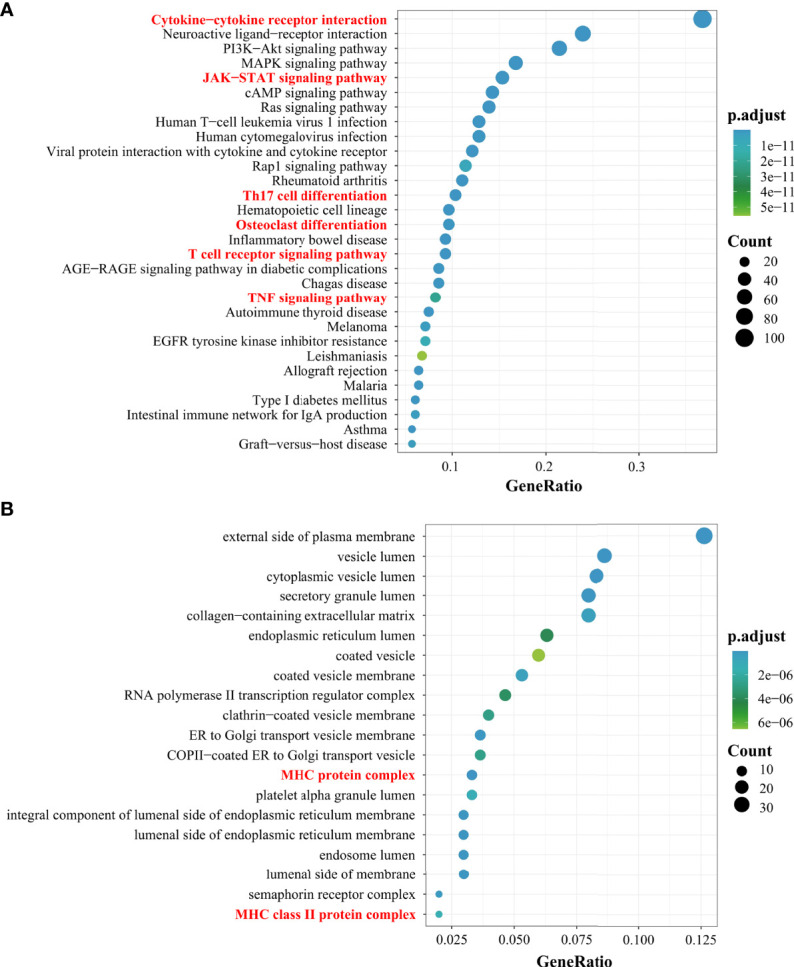
Results of the Gene Ontology (GO) and Kyoto Encyclopedia of Genes and Genomes (KEGG) analyses. The top 30 KEGG pathway enrichment candidate targets of the target genes **(A)**. Pathways with significant changes (FDR < 0.05) were identified. The vertical coordinates represent the KEGG pathway with significant enrichment, and the horizontal coordinates represent the gene ratio which refers to the ratio of enriched genes to all target genes. The top 20 GO enrichment candidate targets of the target genes **(B)**. The color of the bubble graph indicates the categories of “cellular components” in the GO of the target genes (FDR < 0.05), and the horizontal coordinates represent the gene ratio which refers to the ratio of enriched genes to all target genes.

In the article, we used bioinformatics analysis methods (**Supplementary 1**) to integrate multiple databases for the screening of different genes involved in PMOP. Next, we performed enrichment analysis of the Kyoto Encyclopedia of Genes and Genomes pathway. Of the top 30 pathways, some pathways associated with immunity in PMOP were enriched (e.g., cytokine–cytokine receptor interaction, JAK-STAT signaling pathway, Th17 cell differentiation, OC differentiation, T-cell receptor signaling pathway, and TNF signaling pathway) (**Figure 1A**). The top 20 Gene Ontology enrichment candidate targets of the different genes associated with immunity in PMOP are shown in **Figure 1B**. There was a pathologic crosstalk of core cytokine networks involved in PMOP and immunity. Gene Ontology functional enrichment analysis of common differentially expressed genes in PMOP and immunity was performed, including the cellular component. **Figure 1B** shows the enrichment of the MHC-II protein complex.

**Figure 2:**
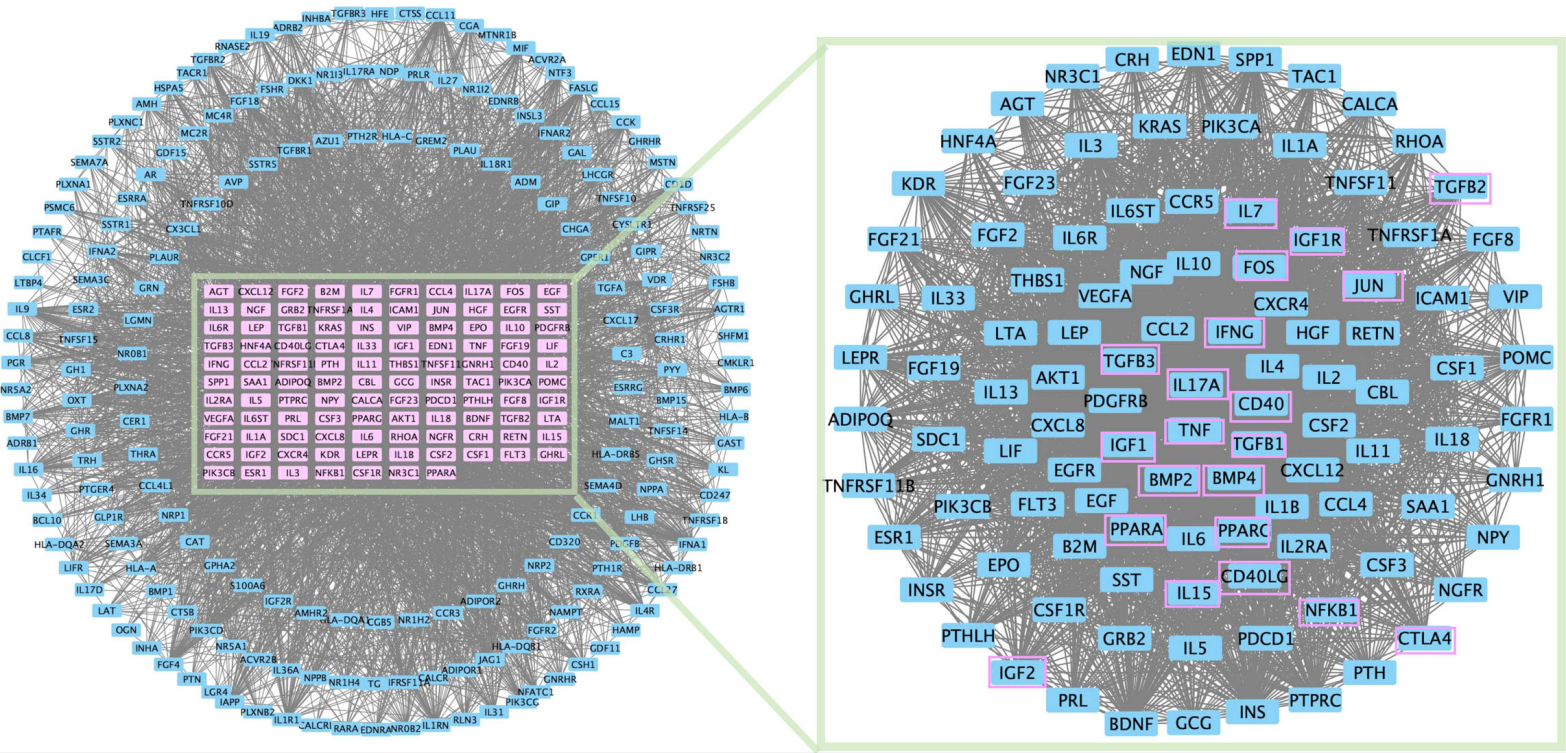
PPI network topology analysis was conducted for common differential genes in PMOP differential genes and immunity genes.

In addition, protein–protein interaction network topology analysis was conducted to identify common differentially expressed genes in PMOP and immunity genes (**Figure 2**). IL-17A, TNF, and IFN are derived from T_E_; IGF is associated with the membrane integrity of phagolysosomes; PPAR is associated with the differentiation of BMSCs into adipocytes; CD40L can be expressed only on activated T cells; and cytotoxic T-lymphocyte-associated protein 4 (CTLA4) can provide inhibitory information for T-cell activation.

**Figure 3:**
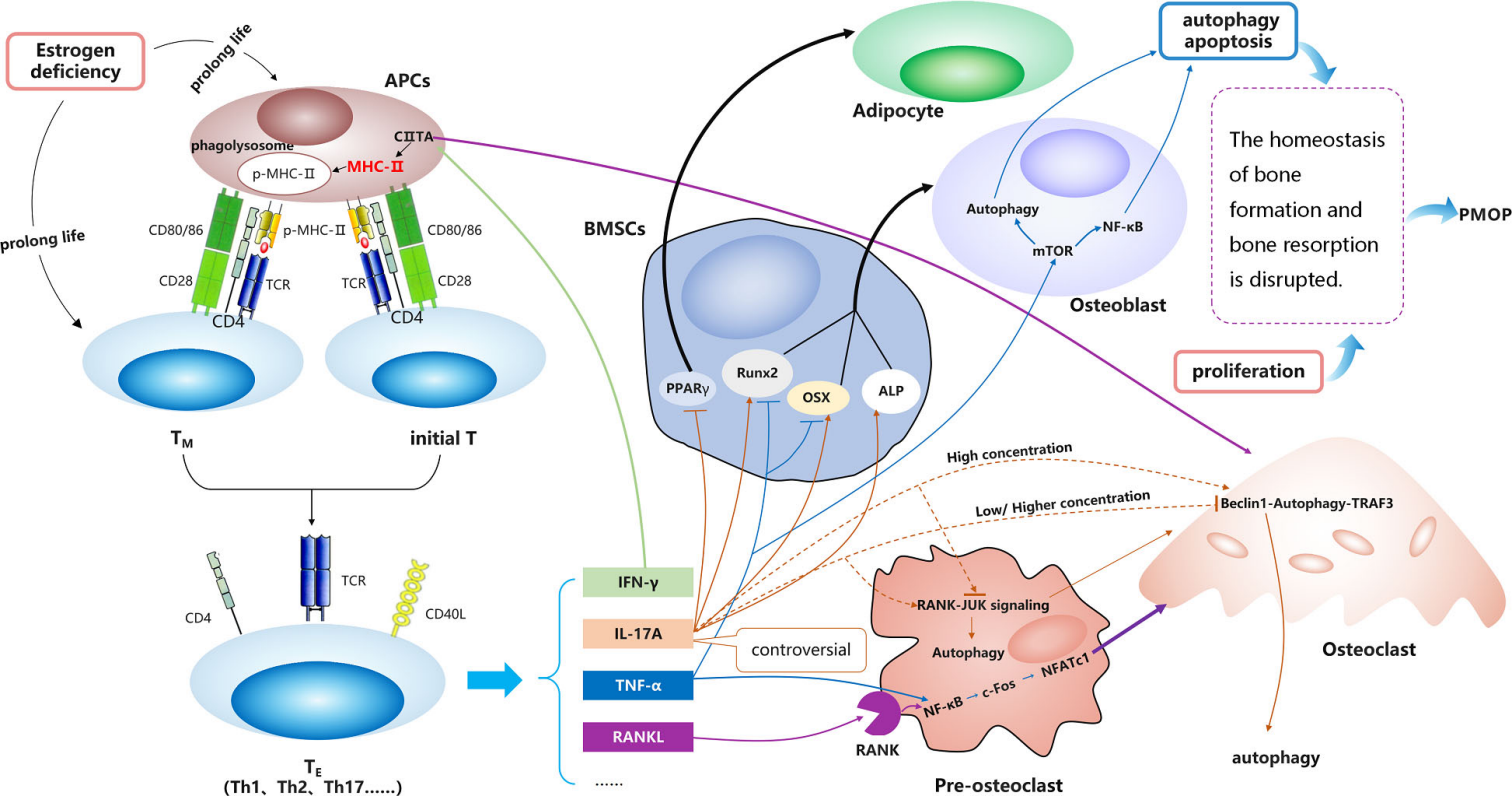
The relationship between MHC-II and PMOP.

These results indicate that MHC-II molecules may promote OC differentiation and play crucial roles in the development of PMOP. They mainly act through various cytokines produced by T_E_ cells, some may act on osteoblasts, and some may act on osteoclasts (**Figure 3**).

## Conclusion

This review focused on the MHC-II molecular protein presentation pathway. The phagolysosomal membrane integrity and CIITA are critical to the MHC-II molecular protein presentation pathway. The evidence suggests that MHC-II molecules play a key role in OC differentiation, providing a new direction for revealing the pathological mechanism underlying the development of PMOP. Such knowledge may provide potential therapeutic targets for the prevention and treatment of PMOP. According to the network pharmacology analysis, the common differential genes in PMOP differential genes and immunity genes were found. However, which genes are upregulated and which are downregulated and their specific roles in bone immunity need to be further explored.

## Author contributions

The manuscript of this review was composed by XW, XZ, Y-DH, XD, JW, HY, SW, YX, ZZ, LW, Y-FH, QL, XT, JZ, HZ, MM, WG, and ZY under the guidance and advice of XL. All authors contributed to the article and approved the submitted version.

## Funding

This work was funded by the National Natural Science Foundation of China (No. 82074461), the Natural Science Foundation of Fujian Province (No. 2020J01719), and the Chen Keji Integrated Chinese and Western Medicine Development Fund-supported projects (No.CKJ2021012).

## Acknowledgments

We would like to thank the two reviewers for their helpful suggestions which helped us to improve the manuscript.

## Conflict of interest

The authors declare that the research was conducted in the absence of any commercial or financial relationships that could be construed as a potential conflict of interest.

## Publisher’s note

All claims expressed in this article are solely those of the authors and do not necessarily represent those of their affiliated organizations, or those of the publisher, the editors and the reviewers. Any product that may be evaluated in this article, or claim that may be made by its manufacturer, is not guaranteed or endorsed by the publisher.
