# Supplementary material for: Role of the major histocompatibility complex class II protein presentation pathway in bone immunity imbalance in postmenopausal osteoporosis
Source: Front Endocrinol (Lausanne). 2022 Aug 11;13:876067. doi: 10.3389/fendo.2022.876067 (PMC9402988; doi:10.3389/fendo.2022.876067)
Supplement: Supplementary file 1 [file DataSheet_1.pdf]

## METHODS OF BIOINFORMATICS

### Differential Genes Screening of Postmenopausal Osteoporosis (PMOP) and Immunity Genes

Differential Genes Screening of PMOP were obtained by GEO, DisGeNET, DrugBank, GeneCards, Pharmgkb.The original file GSE56116 screened on GEO database, were processed by a robust multiarray average algorithm with normalization of matrix data, and the relevant data were filtered using the Limma package to analyze the chip data twice, combining the *P*-value, and the difference multiple. The screening conditions for significantly differentially expressed genes were $P < 0.05$ with a $|\log 2 \text{ (fold change)}| > 0.05$. The screening results of different genes in all databases are shown in Table S1. Then, the differential genes screened by multiple data were merged and the duplicate genes were removed. After this, the final genes are the total differential genes of PMOP. Immune related genes were obtained from IMMPORT database. Intersection genes are common genes between differential genes of PMOP and immunity genes.

### Enrichment Analysis of Kyoto Encyclopedia of Genes and Genomes (KEGG) Pathway of Intersection Genes

The KEGG database (https://www.kegg.jp/) was used to identify the function and biological correlation of candidate target genes. Cluster Profiler R package was used to visualize the KEGG pathway data. The pathways which exhibited significant changes with an false discovery rate (FDR) < 0.05 were selected for further analysis, and the top 30 KEGG pathways were selected.

### Gene Ontology (GO) Functional Enrichment Analysis of Intersection Genes

The Gene Ontology database (GO, http://geneontology.org/) was used to identify biological mechanisms from high-throughputgenomic or transcriptome data. Cluster Profiler R package was used to visualize the GO function data. The functional categories were enriched within genes (FDR < 0.05), and the top 20 GO functional categories were selected.

### Protein–Protein Interaction (PPI) Core Network Construction of Intersection Genes

The intersection genes used to construct PPI network through STRING 11.0 database (https://www.string-db.org/) (highest confidence > 0.4). Then, using the network topology analysis plugin CytoNCA and filtering with Betweenness Centrality (BC), Degree Centrality (DC), Closer Centrality (CC), Eigenvector Centrality (EC), Local average connectivity-based method (LAC) and Network Centrality (NC), key genes were identified in the PPI network.

**Table S1**. Summary of PMOP differential genes and Immunity Genes

| | Database | The number of genes | Website |
|---|---|---|---|
| PMOP differential genes | GEO (GSE56116) | 971 | https://www.ncbi.nlm.nih.gov/ |
| | Disgenet | 171 | http://www.disgenet.org/ |
| | DrugBank | 103 | https://www.drugbank.com/ |
| | GeneCards (score⩾1) | 896 | https://www.genecards.org/ |
| | Pharmgkb | 15 | https://www.pharmgkb.org/ |
| Immunity Genes | IMMPORT | 1173 | https://www.immport.org/ |
